# Copper Exposure Affects Anti-Predatory Behaviour and Acetylcholinesterase Levels in *Culex pipiens* (Diptera, Culicidae)

**DOI:** 10.3390/insects13121151

**Published:** 2022-12-14

**Authors:** Nermeen R. Amer, Sharon P. Lawler, Nawal M. Zohdy, Aly Younes, Wael M. ElSayed, Guillaume Wos, Samah Abdelrazek, Hind Omer, Richard E. Connon

**Affiliations:** 1 Entomology Department, Faculty of Science, Cairo University, Giza 12613, Egypt; 2 Institute of Nature Conservation, Polish Academy of Sciences, 31-120 Krakow, Poland; 3 Entomology and Nematology Department, University of California Davis, Davis, CA 95616, USA; 4 Department of Anatomy, Physiology & Cell Biology, University of California Davis, Davis, CA 95616, USA

**Keywords:** mosquito, cues, toxicity, acetylcholine, enzyme, copper sulphate

## Abstract

**Simple Summary:**

Interaction between natural and anthropogenic stressors, such as contaminants and predators, could jointly account for potential ecological risk to organisms. This study quantified the combined effect of copper and/or predation cues (non- consumptive predation) on the anti-predatory behaviour of *Culex pipiens* larvae (swimming distance and speed). As well, we tested the ability of mosquito larvae to escape predation by dragonfly larvae. We also measured the long-term effect of copper on an enzyme important to the nervous system, acetylcholine esterase (AChE) for two successive generations. Copper reduced the movement and velocity of *Cx. pipiens* larvae, even at levels regarded as environmentally safe. Interestingly, copper showed some stronger effects in the second generation than the first one. Copper acted as an AChE inhibitor at 500 µg L^−1^. There was no significant effect of copper on the ability of larvae to escape from the direct predation of dragonflies in the laboratory, where most were consumed rapidly. However, the behavioural and neurological changes documented could result in *Cx. pipiens* larvae being more vulnerable to predation in natural habitats. Copper likely bioaccumulated and was passed on in eggs, and/or had other maternal or gene expression effects, resulting in harmful effects on offspring.

**Abstract:**

Copper is an essential metal that occurs chronically in the environment and affects the development and physiology of aquatic insects. In excess amounts, it can impair their nervous system and behaviour. We tested the anti-predatory behaviour of *Cx. pipiens* larvae after seven days exposure with several concentrations of copper up to 500 mg L^−1^. We measured responses to non- consumptive (predation cues) and consumptive predation (dragonfly larvae) across two generations. We also tested the accumulated effect of copper on AChE enzyme activity. We exposed half of treated and control larvae to predation cues (water with predator odour and crushed conspecifics) and the other half to water without predation cues. We evaluated total distance moved and velocity. Copper reduced the distance moved and velocity, with stronger effects in the second generation. Copper had no significant effect on larvae eaten by dragonflies. Copper inhibited the AChE enzyme across both generations at 500 µg L^−1^. Copper can affect the nervous system directly by inhibiting AChE activity, and possibly also by impairing the olfaction sensors of the larvae, resulting in larval inability to detect predation cues.

## 1. Introduction

Industrialisation and farming have resulted in higher concentrations of heavy metals such as copper, cadmium, lead and chromium in both terrestrial and aquatic systems [1,2,3,4]. These metals accumulate in organisms’ tissues, leading to acute and chronic effects such as growth reduction, reduced reproduction and developmental aberrations [5]. An ongoing effort in industrialisation and farming is to reduce pollution; therefore, we must alter our vision to understand contaminant effects and use efficient compounds with low or no environmental toxicity.

Copper is widely used in manufacturing and agriculture. It is a primary constituent of algaecides and fungicides in pools and rice fields; it can be used with a concentration up to 1000 µg L^−1^ [6,7]. According to the U.S. Environmental Protection Agency, a copper concentration of 1300 µg L^−1^ is safe for drinking water [8]. Copper pollution has played an important role in reducing the abundance and diversity of aquatic insect communities [9,10,11]. Excess copper impacts the nervous system of aquatic organisms [12] potentially inhibiting acetylcholinesterase (AChE) enzyme activity, a key enzyme in the nervous system for signal transmission [13,14]. Copper is known to affect olfaction and anti-predator behaviour in a variety of insects [15]. In addition, the long-term parental exposure to copper may cause toxic effects on offspring [16] and induce harm to organisms at the physiological and biochemical levels [17]. However, there have not been many studies of copper’s exposure effects or of how copper contamination interacts with insect anti-predator behaviours.

Insects are effective bioindicators of metal exposure in aquatic habitats due to their ability to accumulate metals and possibly other pollutants, with bioaccumulated concentrations mostly reflecting amounts present in their environment [18]. We used *Culex pipiens* as a study organism because of its vast global distribution [19,20]. Moreover, it is a significant vector of pathogens causing many diseases, including West Nile fever [21], periodic lymphatic filariasis [22,23], St Louis encephalitis [24], Japanese encephalitis, Western equine encephalitis and Rift Valley fever [25,26], etc. If copper affects larval mosquito locomotion and their ability to respond to predator cues, this could change the efficacy of natural biological control of mosquitoes. In addition, *Cx. pipiens* is considered to be a relatively tolerant mosquito to poor water conditions [27,28], therefore any toxicological effects will be worrying for other insects.

In this study, we tested the effect of long-term exposure to copper on the anti-predatory behaviour of the larvae of *Cx. pipiens* for two successive generations. Predation is one of the most important factors controlling larval mosquito numbers [29]. Mosquitoes can use predation cues like predator-conditioned water and crushed conspecifics to recognize predators, and in detecting the level of threat [30,31]. Lower activity is often a response to predation danger [32,33], and can cause mosquitoes to reduce their movement and feeding time [31,32,34]. Thus, anti-predator behaviour can be indicated by reductions in the distance moved and velocity of larvae exposed to predation cues. Aquatic media are ideal for dispersion of chemical cues from predators [35]. Prey often sense predators through their signature odour, i.e., a kairomone. A kairomone is a chemical substance released by the predator, and received by the prey, which is adaptively favorable to the prey but not the predator [36]. Prey that can detect kairomones, can potentially evade predators [36]. Crushed conspecifics also produce strong cues indicating predation threat, even in the absence of predator-specific cues [37].

We aimed to determine if *Cx. pipiens* larvae could recognise the presence of a predator threat (dragonfly-conditioned water with crushed conspecifics cue added), and whether exposure to various concentrations of copper could affect their movement behaviour, with or without predator cues. We also tested copper’s effect on the mosquitoes’ ability to escape direct predation. A further aim was to assess whether copper acts as a neuro-inhibitor metal on mosquito larvae, mechanistically inhibiting AChE activity at tested concentrations. We hypothesised that elevated concentrations could impair mosquito responses to predators. We designed this study as a long-term exposure across two successive generations to test potential bioaccumulation effects of copper on anti-predatory behaviour.

## 2. Materials & Methods

### 2.1. Prey Collection and Initial Rearing

*Culex pipiens quinquefasciatus* larvae were supplied by the Sacramento-Yolo Mosquito and Vector Control District. The strain originated from Merced, CA, USA sometime in the 1950’s, and has been maintained as a ‘susceptible’ strain, unexposed to toxicants as a standard model in environmental toxicity testing. We reared them in large enamel trays (40 cm × 25 cm) each filled with 1 L of reconstituted deionised water (RDiW) under insectary room conditions at 25 ± 0.5 °C, ~80% humidity and 18:6 h light: dark photoperiod (Darwin insect growth chamber, Model EFA3MB1, St. Louis, MO, USA). The reconstituted deionised water was prepared by adding 0.06 g MgSO_4_, 0.06 g CaSO_4_·2H_2_O, 0.096 g NaHCO_3_ and 0.004 g KCl separately to 1 L deionised water, stirred and kept uncovered overnight [38]. Following a protocol used by the Sacramento-Yolo Mosquito and Vector Control District, California, USA, we prepared a larval diet using 45% each of ground alfalfa and ground fish food, plus 5% of each nutritional yeast and ground liver. We added a daily 0.02 g of larval food to each tray of the younger larvae, increasing the amount to 0.04 g as larvae grew. The initially supplied larval mosquitoes (third and fourth instar) were reared until they emerged as adults. We kept these in cages (30 cm × 30 cm × 30 cm) under ambient laboratory conditions. Emerged adults were provided with a sponge soaked in 10% sugar solution in each cage. Heparinised sheep blood meal (Hemostat Laboratories, Dixon, CA, USA) was provided for four-day-old females to obtain F1 eggs, by using a small beaker covered with plastic parafilm inverted on the top of the cage. We provided oviposition cups (12 (∅) cm × 7 (height) cm filled with 200 mL RDiW). Resulting egg rafts were reared in the large enamel rearing trays until hatching, then they were transferred to start the experiment.

### 2.2. Preparation of Copper Solutions

We prepared different concentrations of copper sulphate pentahydrate (CuSO_4_·5H_2_O; Sigma Aldrich, CA; purity: 99.9%): 0 (control), 125, 250 and 500 µg L^−1^. We chose these copper concentrations to be environmental relevant and under the safe limit of copper in drinking water [8,39,40]. The stock solution of 10 g L^−1^ of CuSO_4_·5H_2_O was prepared by adding 0.1 g of CuSO_4_·5H_2_O to 10 mL RDiW, mixed thoroughly in a volumetric flask. We prepared the four concentrations of CuSO_4_·5H_2_O, 0 (control), 125, 250 and 500 µg L^−1^ in RDiW from the stock solution for the first and second generations. We saved four samples each of 30 mL of control water and each concentration at 4 °C immediately after preparation for chemical analysis. We used nominal concentrations of CuSO_4_·5H_2_O to obtain expected concentrations of dissolved copper of 0, 31, 63 and 127 µg Cu L^−1^ corresponding to 0, 125, 250 and 500 µg L^−1^ of CuSO_4_·5H_2_O, respectively. Expected concentrations were confirmed by the Interdisciplinary Center for Plasma Mass Spectrometry, UC Davis, using an Agilent 8900 Triple Quadrupole mass spectrometer (Agilent Scientific Instruments, Santa Clara, CA, USA).

### 2.3. Predator Rearing and Preparation of Predator Cues and Predator Water

Dragonfly immatures (naiads) had been collected from outdoor mesocosms, which were established several months earlier for colonisation and rearing aquatic predators at Putah Creek, University of California Davis. Mesocosms were 1325 L polyethylene tanks filled with well water, which contained sand and compost substrate, and rooted macrophytes collected from a nearby stream. We prepared predator water by individually rearing Libellulidae dragonflies (with size range 8–10 mm) in cups contain ~50 mL of well water. Each predator was fed with daily five mosquito larvae (4th instar). Dragonfly mortality was checked daily during the experiment. According to Ferrari et al. [34], who detected significant effects from cues, we prepared predator cues from crushed conspecific organisms by crushing five fourth instar *Cx. pipiens* larvae with a mortar and pestle and diluting the paste in 5 mL RDiW.

### 2.4. Experiment 1: Anti-Predatory Behaviour Tests

We reared larvae with the same procedure described for initial rearing, with some alterations for treatment exposures. We established duplicate rearing polypropylene plastic pans of larvae at each concentration of copper and controls under controlled environmental chamber conditions of 25 ± 2 °C and 18:6 h light: dark photoperiod (Darwin insect growth chamber, Model EFA3MB1, St. Louis, MO, USA). The pans with diameter and height of 25 cm × 15 cm, filled to 5 cm deep. Each pan contained 1 L of the corresponding CuSO_4_·5H_2_O concentration and 100 first instar larvae (<24 h old) drawn randomly from different egg rafts (>three egg rafts) after hatching. Larvae were exposed to the assigned concentrations or control water for seven days after hatching in each generation. We renewed ~90% of the solutions every 48 h to keep the concentration constant. We checked larval mortality every day and removed dead organisms from the vessels but without counting them due to time constraints.

After seven days of larval exposure to each treatment, we prepared replicates of two kinds of petri dishes (P1 and P2, 9 cm diameter); (P1) filled with 30 mL of predator rearing water plus 1 mL of crushed conspecific and the other (P2) filled with 30 mL RDiW as a negative control. We performed replicated recordings of 26 to 30 larvae per control and each copper exposure treatment (depending on availability). Half of these number (n = 13–15) were exposed to predator rearing water with crushed conspecific cues (P1) and the other half to RDiW water only (P2). For the 1st generation, the number of exposed larvae to predator water plus crushed conspecifics were (15, 14, 15, and 13 larvae corresponding to control, 125, 250, and 500 µg L^−1^, respectively), while those exposed to RDiW were (15, 14, 15, and 14 larvae corresponding to control, 125, 250, and 500 µg L^−1^, respectively). For the 2nd generation, the number of exposed larvae to predator water plus crushed conspecifics were (13, 13, and 14 larvae corresponding to control, 125, and 250 µg L^−1^, respectively) while those exposed to RDiW were (13, 13, and 15 larvae corresponding to control, 125, and 250 µg L^−1^, respectively). We kept larvae without food for 24 h to standardise their hunger levels before video recording. For each recording, two larvae were picked randomly from the rearing trays of controls and each concentration. We added one larva in the center of each dish using a pipet, allowing three min to acclimate. We recorded video in MPEG-2 format for five min using a Panasonic^®^ black and white CCTV camera focused on the two petri dishes using a tripod. We analysed videos using Ethovision XT 6.1 Software (Noldus Information Technology Inc., Leesburg, VA, USA) to determine the distance moved (cm) and velocity (cm s^−1^). The two-dimensional movement was determined by analysing the movement relative to the center-point of each organism’s body. We analysed velocity by measuring the mean speed only during the times when larvae were moving. Predator water and crushed conspecifics were renewed hourly. Using these videos, we captured photos and measured the whole-body length of *Cx. pipiens* larvae from top of the head to the end of the abdomen using Tracker Software.

The remaining larvae in the control pans and each copper concentration were reared for the second generation. Pupae and emerged adults were kept in separate cages (30 cm × 30 cm × 30 cm) for the control and each concentration using the same initial rearing. A minimum of 10 adults were kept in each cage for control and each copper concentration. At least three egg rafts were used for controls and each copper concentrations to continue the second-generation toxicity and behaviour experiments. We performed the same procedure for the second-generation larvae. We omitted the behaviour test at 500 µg L^−1^ of the second generation as mosquitoes reared at this concentration were too small to be detected by Ethovision software.

### 2.5. Experiment 2: Direct Predation Effect

We prepared three replicates of rearing polypropylene plastic pans (25 (∅) cm × 15 (height) cm) at control and each copper concentration under controlled environmental chamber conditions of 25 ± 2 °C and 18:6 h light: dark photoperiod (Darwin insect growth chamber, Model EFA3MB1, St. Louis, MO, USA). Each pan contained 100 first instar larvae (<24 h old). Larvae were exposed to the corresponding concentration for seven days after hatching in each generation. We reared larvae with the same procedure described for initial rearing, with some additions. We renewed ~90% of the solutions every 48 h to keep the concentration constant. We checked larval mortality every day and removed any dead organisms from the vessels. Pupae and emerged adults were kept in separate cages (30 cm × 30 cm × 30 cm) for the control and each concentration using the same initial rearing procedure to continue the second-generation toxicity and direct predation experiment.

After seven days of larval exposure to the corresponding copper concentration, we prepared five replicates of polyethylene plastic cups (12 (∅) cm × 7 (height) cm) for control and each copper concentration. Each replicate contained 200 mL of the corresponding copper concentration and 10 *Cx. pipiens* larvae picked randomly from the relevant rearing pans. After five min, we introduced one Libellulidae dragonfly larva to each cup. Photos of all replicates were taken at the start of the experiment for length measuring. We measured the whole body length of both mosquito and dragonfly larvae from top of the head to the end of the abdomen using Tracker Software. Mosquito and dragonfly larvae were kept without food for 24 h before the experiment to standardise their hunger levels. We recorded the number of mosquito larvae eaten after 10 min and 60 min. We performed the same procedure for the second-generation larvae.

### 2.6. Experiment 3: AChE Activity Assessment

To assess the effect of different copper concentrations on cholinesterase enzymatic activity of *Cx. pipiens* larvae across two successive generations, we prepared three replicates each of 1 L of copper sulphate pentahydrate concentrations (0 (control), 125, 250 and 500 µg L^−1^) as described above. We reared the replicate groups of larvae at each copper concentration and control under laboratory-controlled conditions of 25 ± 2 °C and 18:6 h light: dark photoperiod. For each replicate, we exposed 100 first instar larvae (<24 h old) drawn randomly from different egg rafts (≥5) in a polypropylene plastic pan with diameter and height of 25 cm and 15 cm, respectively. We added 0.02 g of larval food to each container, increasing the amount to 0.04 g as larvae grew. About 90% of the solution was renewed every 48 h. We removed any dead organisms from the vessels. After seven days, we preserved three replicates each of ~25 mg of whole larval tissues (10–20 larvae, 2nd–4th instar depending on copper concentration) from each copper concentration at −20 °C. Remaining larvae were kept and reared until adult emergence under the same conditions. Emerged adults from the first generation were kept in cages (30 cm × 30 cm × 30 cm, as described above) to continue with the second-generation experiment.

We used Abcam acetylcholinesterase assay kits (ab138871, Cambridge, MA, USA). Briefly, tissues of each replicate were washed and homogenized using cold PBS, then centrifuged at 2500 rpm for 10 min to use the supernatant. Acetylthiocholine- reaction mixture and acetylcholine standard were prepared and loaded to 96 well plate with the samples following to Abcam protocol. Five µL of (1 µM) Donepezil inhibitor was added to each sample well. Seven inhibitor concentrations (0, 3, 10, 30, 100, 300 and 1000 µg ml^−1^) (run in duplicate) were used to construct standard dose–response curves. The plate was incubated in the dark for 30 min before being read by Infinite m200 plate reader at 410 nm, I-control^®^ software version 2.0.10. We omitted the results from 250 µg L^−1^ due to a mistake during loading the well plate.

### 2.7. Statistical Analysis

We ran generalized linear models for each response variable: distance, velocity, number of larvae eaten and AChE concentration. Distance and velocity were both continuous variables (Gaussian distribution) and were squared-root transformed to meet normality assumptions. For these two variables, the model included copper concentration, predator cue (presence vs. absence of a predator cue) and their interaction; copper concentration was considered as a categorical variable. Because copper affected larval length, and length of larvae correlated with distance and velocity (Figure S1), we added length of larvae as a covariate in the model. We ran two separate models, one for each generation. Number of larvae eaten was count data (Poisson distribution) and the model included duration of predation (10 min vs. 60 min), copper concentration and their interaction. For each copper concentration, we had five replicates which was added as a random factor. We ran two models, one for each generation. Finally, acetylcholine concentration was a continuous variable (Gaussian distribution) and the model included copper concentration, generation and their interaction as predictors. For each copper concentration, we had three replicates which was added as a random factor. Each model was fitted using the function ‘glmmTMB’ (‘glmmTMB’ package; [41]) in R (R Core Team, 2013; RStudio Team, 2015) and *p*-values were computed using Wald chi-square test (Wald X^2^) implemented in the ‘car’ package [42]. We used pairwise comparisons as post-hoc tests (*t*-test) and computed the least square mean (LSM) for each response variable in response to each factor using the “Ismeans” function (emmeans package, [43]). In addition, for each model, we computed partial eta-squared (η2) to estimate the proportion of variance explained by each predictor excluding the others (Etasq function; R package heplots; [44]).

## 3. Results

### 3.1. Effects of Copper and Predation Cues on Behaviour

**Figure 1:**
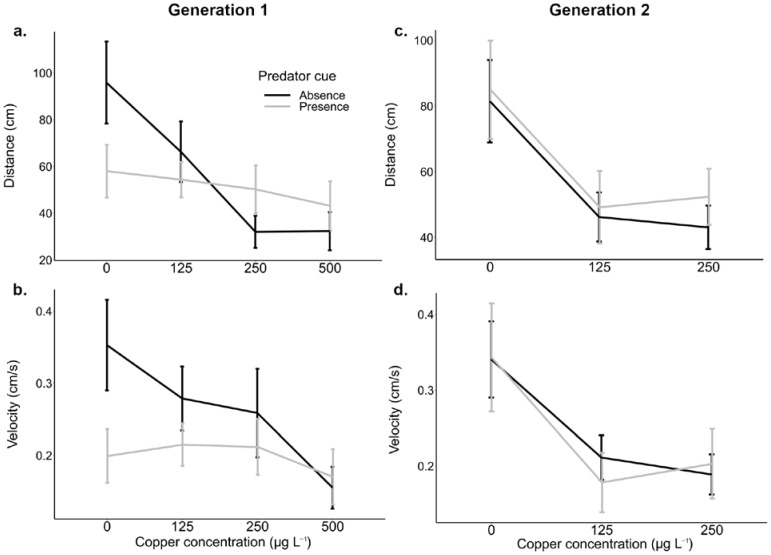
Effects of predation cues and copper concentration exposures on distance and velocity of movements by two generations of *Culex pipiens* larvae. Panels (**a**,**c**) are mean distances for the first and second generations, (**b**,**d**) are mean velocities; bars are standard errors. Connecting lines are included as a visual aid only.

The effects of copper and predation on distance and velocity for both generations are shown in Table 1. Copper inhibited the distance moved and the movement velocity by larvae in both generations. For the first generation of larvae exposed to combination of copper and predator cues, we found significant effects of copper (η2 = 16.6% of variance explained) comparing to larvae swimming longer distances without copper (Table S1). The interaction copper × predation (η2 = 9.43%) was also significant (Figure 1), in the absence of copper, larvae swam longer distances in the absence of a predator cue compared to those exposed to a predator cue (t-ratio = 2.224, df = 107, *p* = 0.028). An increase in copper concentration further decreased the distance swum by larvae in the absence of a predator cue, whereas in the presence of a predator cue the distance swum by larvae was less affected by an increase in copper concentration. Velocity was significantly affected by both copper (η2 = 8.94%) and predator cue (η2 = 4.37%); with higher velocity without copper and in the absence of a predator cue (Figure 1b, Table 1). The interaction was not significant. By measuring and analysing larval length, we noticed that copper inhibited larval growth significantly (Wald X^2^ = 5652; df = 3; *p* < 0.001) at all copper levels (Table S2).

For the second generation, the effect of a predator cue on distance and velocity was no longer significant. We found stronger effects of copper on both distance (η2 = 42.8%) and velocity (η2 = 39.9%) (Figure 1c,d, Table 1) with a higher proportion of variance explained compared to the first generation. The distance swum by larvae and velocity were significantly higher without copper (Figure 1c,d, Table S3) and no difference was found in distance and velocity when comparing low (125 µg L^−1^) with medium (250 µg L^−1^) copper concentration (Table S3). Larval growth has been significantly inhibited by copper (Wald X^2^ = 8323; df = 2; *p* < 0.001) at all copper levels (Table S3).

The mean larval length of controls was 6.97 ± 0.45 and 6.58 ± 0.28 mm while at the highest concentration (500 µg L^−1^) lengths averaged 2.55 ± 0.47 mm and 1.98 ± 0.08 mm for the first and second generations, respectively. Mortality patterns of mosquito larvae seemed generally similar to our prior work at the same concentrations. Our earlier study showed that copper reduced *Cx. pipiens* survival of the first generation to 12 and 48% at 250 and 500 µg L^−1^, respectively, while in the second generation, copper reduced *Cx. pipiens* survival to 26 and 82% at 250 and 500 µg L^−1,^, respectively [45].

### 3.2. Effect of Copper on Escaping Behaviour

**Figure 2:**
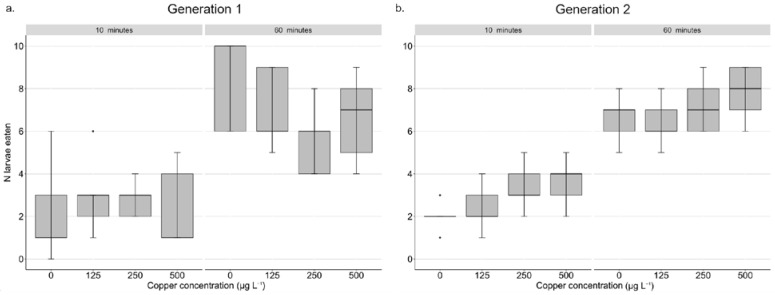
Effect of seven days of copper concentration exposures on the number of *Culex pipiens* larvae eaten per dragonfly after 10 min and 60 min across two successive generations. Boxes shows the average number of larvae eaten for each copper concentration and generation (N = 5 per copper concentration). Panels (**a**) is the average number of larvae eaten in generation 1, (**b**) is the average number of larvae eaten in generation 2. Error bars are included, and dots represent individuals.

Next, we tested whether an increase in copper concentration affected the ability to escape a dragonfly predator. We did not find significant effect of copper concentration on the number of larvae eaten by the predator and results were similar for both generations (Figure 2, Table S4). All dragonflies survived in the predation experiment.

### 3.3. Effects of Copper on AChE Concentration

**Figure 3:**
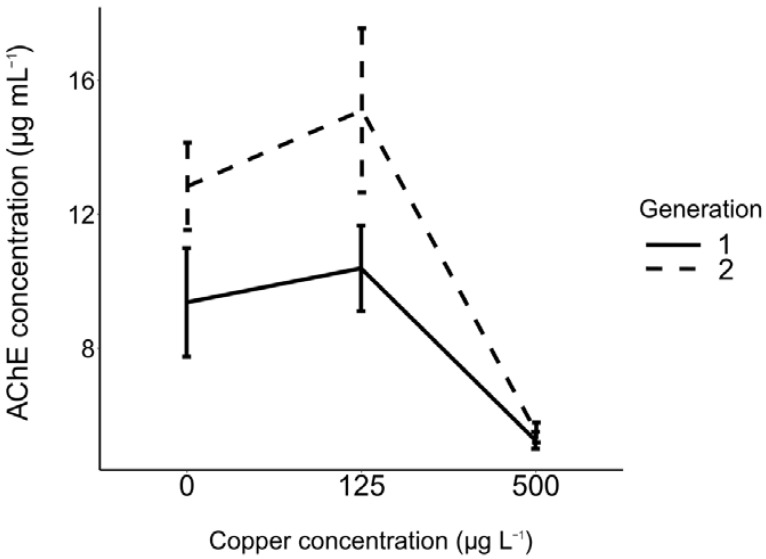
Effect of different concentrations of copper exposure (0, 125 and 500 µg L^−1^) on acetylcholinesterase (AChE) activity across two successive generations of *Culex pipiens* larvae.

AChE concentration was significantly affected by copper concentration and explained a large proportion of variance (Wald X^2^ = 44.6, Df = 2, *p* < 0.001, η2 = 71.2%). AChE concentration was higher under control (0 µgL^−1^) and low copper concentration (125 µg L^−1^) with no significant difference between these two conditions, but it decreased significantly under high copper concentration (500 µg L^−1^) (Figure 3, Table S1). There was also a significant generational effect (Wald X^2^ = 8.78, Df = 1, *p* = 0.003, η2 = 32.8%) with a higher AChE concentration found in the second generation (Least Square mean [LSM] Acetylcholine generation 1 = 8.34 µg mg^−1^; AChE generation 2 = 11.15 µg mg^−1^) (Figure 3).

## 4. Discussion

Copper contamination has been shown to affect animal physiology, locomotion, and olfaction [29,46,47]; thus, it could affect how invertebrates respond to predator cues. Our results revealed strong reductions in larval mosquito locomotion due to prior copper exposure and/or predator cues, and effects of copper on AChE. Effects of copper were stronger in a second generation exposed to copper than in the first. Predation cues are not only useful in predator recognition, but their concentration also determines the risk level and degree of anti-predator response needed [34,48]. From our overall literature review for this study, we believe that this is the first study to address the combined effects of long exposures to copper and predation cues as factors impacting the anti-predatory behaviour of mosquito larvae, although these factors have been examined separately.

In comparison to copper-free controls, copper decreased the distance moved and the movement velocity of *Cx. pipiens* in both generations, with effects on distance beginning at concentrations of 250 µg L^−1^ in the first generation and 125 µg L^−1^ in the second generation, and effects on velocity at all levels in the second generation. Hence, these findings reveal the maternal effect of copper on offspring. These results also correspond with a single-generation study that showed that copper treatment likely reduced the escape behaviour of second and third instars of *Anopheles stephensi* mosquito larvae that were exposed to guppies (*Poecilia reticulata*) [29]. Reduced movement is often a defense response because many predators are drawn to prey movement, so the effects of reduced movement from copper and predator cues on prey survival may depend on whether larvae are hidden or exposed.

On the other hand, several studies have evaluated the impact of predator cues alone on behaviour, revealing significant effects of predation cues on mosquito larvae behaviour. A study on the second and third instars of *Culiseta longiareolata* mosquito larvae [49] also identified a reduction in their activity and changes in their feeding behaviour when exposed to the same alarm cues used in the present study. Similarly, *Culex restuans* larvae responded effectively to alarm cues [36]. In another study, *Aedes* larvae exposed to predators increased resting at the air–water interface or increased flexing [33]. In contrast, studies of *Anopheles gambiae* or *Culex quinquefasciatus* larvae showed no detectable responses to similar cues [49,50].

Due to the scarcity of literature dealing with multiple stressors, we are unable to directly compare the combined effects of copper and cues with other mosquito studies. Interestingly, in this study, the interaction between copper and predation cues had a significant effect only on the distance moved in the first generation. These results could indicate copper’s ability to eliminate predator cue effects, or in another words, copper might inhibit the larval ability to detect predation cues. Excess levels of copper are also known to impact other invertebrate and vertebrate species [16,51,52,53]. Copper affected the caddisflies *Hydropsyche morose* by increasing their vulnerability to predation by stoneflies [54]. Another study pointed out the inhibition effect of copper on the burrowing behaviour of two benthic invertebrates, the polychaete *Nereis diversicolor* and the bivalve *Scrobicularia plana* [52]. Olfactory inhibition could underlie these effects: studies on the effect of copper on fish behaviour revealed that copper can cause olfactory deterioration [55,56]. For example, copper at a low concentration of 2 µg L^−1^ greatly impaired the olfaction of juvenile Coho salmon [57,58]. In another study, copper at concentration of 1.85 µg L^−1^ rendered *Lithobates sylvaticus* tadpoles more vulnerable to dragonfly predation [58]. Since olfaction is an important source of information that many aquatic prey and predators depend on [36], our data are consistent with copper impeding *Cx. pipiens* larvae from sensing predation cues. However, because copper alone reduced movement in the same way as did predator cues, this calls for further mechanistic studies.

Copper did not affect the ability of larvae to escape from direct predation. Part of this result might be because copper suppressed larval growth, and small larvae cannot swim as fast as larger larvae [59]. The mean larval length of controls was 6.97 ± 0.45 and 6.58 ± 0.28 mm while at the highest concentration (500 µg L^−1^) lengths averaged 2.55 ± 0.47 and 1.98 ± 0.08 mm for the first and second generations, respectively. It is also possible that we collected data at intervals that were too long to detect a difference, as predators had eaten most of the larvae by the end of the experiment. Whether or not copper exposure has indirect effects on mosquito survival, it can directly cause mortality.

In addition to copper’s ability to reduce larval *Cx. pipiens* movement, it also inhibited AChE activity in both generations. AChE level is one of the most significant neuro-behavioural measurements that has been used to confirm mechanistic effects of toxicant exposure in living organisms [60]. Inhibition of AChE could be resulted in post-synaptic acetylcholine receptors and so hyper stimulation which leads to physiological disfunction [60]. It might be due to inhibition of AChE gene expression. However, mode of action of AChE inhibition in insects due to metal exposure still unclear. More generally, some studies have reported that metals could inhibit AChE in vertebrates and invertebrates [12]. Copper is mentioned in a study concerned with the development of AChE inhibitors instead of pyrethroid insecticides to control *Anopheles gambiae* mosquito; a vector of malaria-causing *Plasmodium* [61]. Copper can affect many kinds of animals though; copper exposure at 75 µg L^−1^ for five days resulted in inhibition of AChE activity of the clam *Ruditapes decussatus* [62]. Moreover, copper impaired the swimming behaviour of zebrafish, *Danio rerio*, and also inhibited the activity of AChE at concentration of 6.3 µg L^−1^ after 24 h exposure [60]. In our study, the level of AChE was higher in the second generation due to differences in controls and low concentration exposure, suggesting that this enzyme may have been upregulated to compensate for the negative effect of copper sulphate.

Our study demonstrates that *Cx. pipiens* larvae can be used to indicate the toxicological effects of copper and likely other water pollutants. This is important because few aquatic insects used as models in toxicology, even though they are key members of food webs [63]. *Cx. pipiens* is known to be tolerant to environmental toxicants in poor water conditions and any toxic effect should be worrying regarding other aquatic organisms [20]. Copper, like many other toxicants, can pass to higher levels in food webs up to reaching humans where it accumulates in tissues, resulting in biochemical, physiological defects [64]. Excess amount of copper could be resulted in many human disorders associated with bone health, immune function, and alteration of cholesterol metabolism [65]. While copper may be considered safe or even beneficial to humans in drinking water at levels up to 1300 µg L^−^%*,* it may be toxic to some non-target aquatic organisms at levels below this [66,67]. Copper sulphate, which is used as an aquatic herbicide, algicide, and fungicide at concentrations below the safe limits [6,7], also had sublethal impacts on four non-target organisms, *Daphnia magna*, *Hyalella azteca*, *Chironomus tetans* and *Pimphales promelas* with 48 h LC_50_ of 18.9 ± 2.3, 157.8 ± 28.7, 1136.5 ±138.6 and 19.2 ± 3.1 µg Cu L^−1^, respectively [66]. Another study on the effect of copper on non-target organisms revealed growth inhibition to *Danio rerio* fish with 24 h LC_50_, 72 h LC_50_ and 96 h LC_50_ of 140 (0.098–0.191), 53 (0.039–0.076) and 38 (0.028–0.055) µg Cu L^−1^, respectively [68].

On the other hand, there are uses for copper as a pesticide in situations where non-target effects are less likely, such as in water in small containers [69]. Copper can be used to control mosquitoes in their immature instars in water storage barrels at CuSO_4_·5H_2_O concentrations of 500 µg L^−1^ or above [45,69]. Moreover, cemeteries with stone vases lined with copper can be used to avoid mosquitoes breeding in these sites [70]. Copper has also been formulated as nanoparticles to kill mosquito larvae [71,72]. However, there may be environmental concerns with the use of copper for mosquito control in more natural habitats.

## 5. Conclusions

Despite the importance of insect behaviours and the wide range of insects and metals as neuro-inhibitor contaminants, there is only fragmentary information concerning the multi-stressor effects of metals and other natural stressors on insect behaviour [73]. This study quantified the combined impact of copper and/or predation cues on mosquito behaviour, larval ability to escape from predators and AChE enzyme activity. We can conclude that copper had a significant effect on the movement and velocity of *Cx. pipiens* larvae. Moreover, some effects were stronger in the second generation, possibly due to its bioaccumulation in tissues or damage to embryos. In this study, we determined that copper acts as an AChE inhibitor at copper sulphate pentahydrate concentration of 500 µg L^−1^. These effects could result in *Cx. pipiens* larvae being more vulnerable to predation in the aquatic habitats, but this may depend on whether immobility or escape is the better defense in a particular situation, and on how predators respond to the copper.

## Figures and Tables

**Table 1:** Effects of copper exposure, predator cue and their interaction on distance and velocity for the first and second generations of *Culex pipiens* larvae. Length of larvae was added as a covariate. *p*-values and Wald Chi-squared statistic (in parentheses) for each variable. Significance is indicated by *** *p* < 0.001, ** *p* < 0.01, * *p* < 0.05.

		Generation 1			Generation 2	
		Distance	Velocity		Distance	Velocity
Variables	Df	*p*	*p*	Df	*p*	*p*
Length of larvae	1	<0.001 (23.0) ***	<0.001 (17.3) ***	1	<0.001 (56.4) ***	<0.001 (50.2) ***
Copper concentration	3	0.001 (15.4) **	0.002 (15.0) **	2	<0.001 (60.6) ***	<0.001 (53.7) ***
Predator cue	1	0.595 (0.28)	0.027 (4.87) *	1	0.797 (0.07)	0.134 (2.24)
Copper concentration × predator cue	3	0.007 (12.0) **	0.140 (5.48)	2	0.382 (1.92)	0.562 (1.15)

## Data Availability

The data presented in this study are available in “Raw data” file here.

## Supplementary Materials

The following supporting information can be downloaded at: https://www.mdpi.com/article/10.3390/insects13121151/s1, Figure S1: Linear regression between distance and velocity, and length of *Culex pipiens* larvae for the first (left column) and second (right column) generation after seven days of copper treatment. The Pearson correlation coefficient and significance are indicated in the top left corner of each plot. Distance and velocity were squared- root transformed; Table S1: Least square mean values for distance, velocity and acetylcholine concentrations across two successive generations of *Culex pipiens* larvae for each copper concentration and predator treatment after seven days of copper treatment. / means no data were available; Table S2: pairwise comparisons as post-hoc tests (*t*-test) of the first generation of *Culex pipiens* larvae after seven days of copper exposure; Table S3: pairwise comparisons as post-hoc tests (*t*-test) of the second generation *Culex pipiens* after seven days of copper exposure; Table S4: Effect of copper concentrations, duration of predation by immature Libellulidae dragonflies, and their interaction, on the number of *Culex pipiens* larvae eaten after seven days of copper treatment. Two generations were exposed to several concentrations of copper sulphate or were unexposed. Effect of the random factor is not shown. *p*-values and Wald Chi-squared statistic (in parentheses) for each variable. Significance is indicated by *** *p* < 0.001.
