# Supplementary material for: Copper Exposure Affects Anti-Predatory Behaviour and Acetylcholinesterase Levels in Culex pipiens (Diptera, Culicidae)
Source: Insects. 2022 Dec 14;13(12):1151. doi: 10.3390/insects13121151 (PMC9782022; doi:10.3390/insects13121151)
Supplement: Supplementary file 1 [file insects-13-01151-s001.zip › insects-2064220-supplementary.pdf]

<u>**Supplementary information**</u>

Figure S1. Linear regression between distance and velocity, and length of *Culex pipiens* larvae for the first (left column) and second (right column) generation after seven days of copper treatment. The Pearson correlation coefficient and significance are indicated in the top left corner of each plot. Distance and velocity were squared- root transformed.

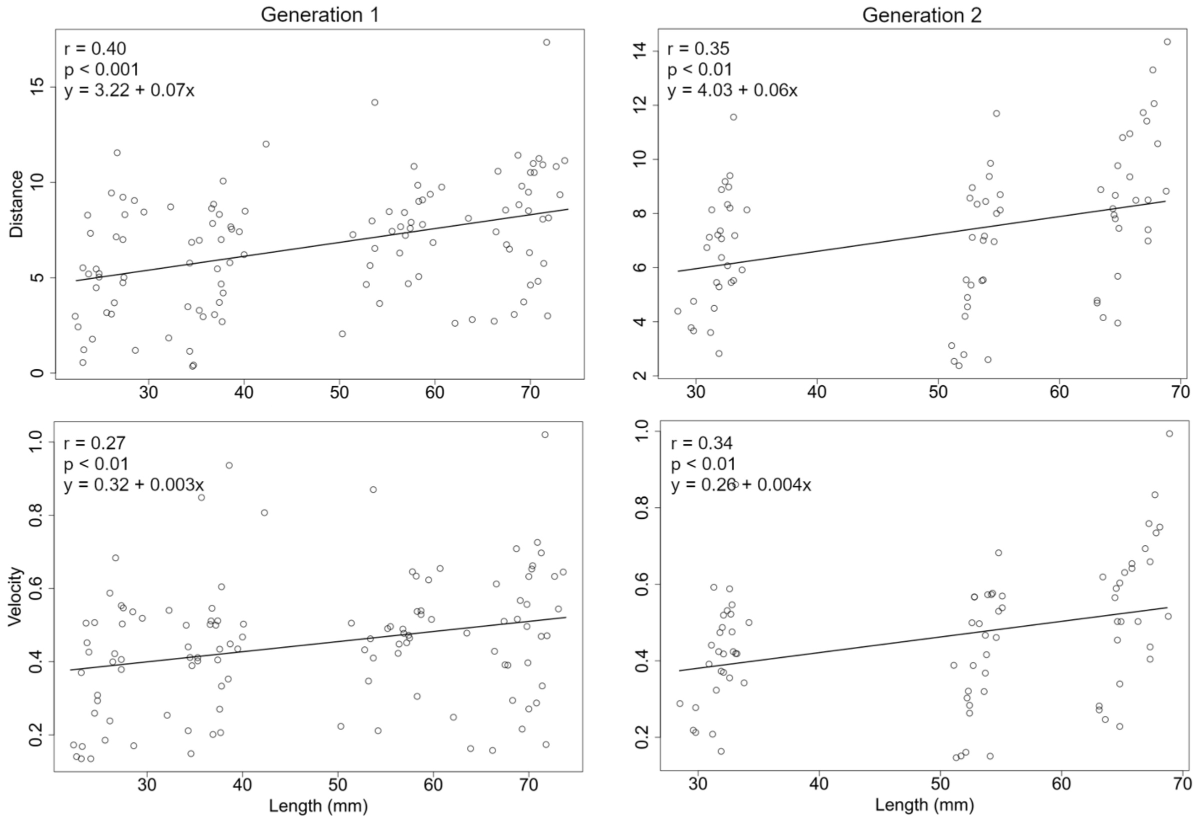

Table S1. Least square mean values for distance, velocity and acetylcholine concentrations across two successive generations of *Culex pipiens* larvae for each copper concentration and predator treatment after seven days of copper treatment. / means no data were available.

Generation 1	**Copper concentration (μg L^{-1})**				**Predator**	
	**0**	**125**	**250**	**500**	**Presence**	**Absence**
**Distance (cm)**	77.00 ± 7.91	60.3 ± 8.34	41.20 ± 7.91	37.60 ± 8.34	51.70 ± 6.06	56.90 ± 6.01
**Velocity (cm/s)**	0.27 ± 0.03	0.247 ± 0.03	0.23 ± 0.03	0.163 ± 0.03	0.200 ± 0.02	0.26 ± 0.02
**Acetylecholine (μg/mg)**	9.37 ± 1.20	10.39 ± 1.20	/	10.4 ± 1.2	/	/
Generation 2						
	**0**	**125**	**250**	**500**	**Presence**	**Absence**
**Distance (cm)**	83.20 ± 7.38	47.70 ± 7.38	47.60 ± 7.38	/	62.00 ± 6.46	56.20 ± 6.38
**Velocity (cm/s)**	0.34 ± 0.03	0.19 ± 0.03	0.19 ± 0.03	/	0.24 ± 0.03	0.24 ± 0.03
**Acetylecholine (μg/mg)**	12.80 ± 1.61	15.10 ± 1.61	/	5.49 ± 1.61	/	/

Table S2. pairwise comparisons as post-hoc tests (t-test) of the first generation of *Culex pipiens* larvae after seven days of copper exposure.

	Generation 1					
**Distance**	**contrast - Copper concentration**	**estimate**	**SE**	**df**	**t.ratio**	**p.value**
	Control - High	2.69	0.78	111	3.42	**0.00**
	Control - Low	0.76	0.77	111	0.98	0.75
	Control - Medium	2.40	0.76	111	3.14	0.01
	High - Low	-1.92	0.80	111	-2.41	0.08
	High - Medium	-0.28	0.78	111	-0.36	0.98
	Low - Medium	1.64	0.77	111	2.10	0.15
**Velocity**	**contrast - Copper concentration**	**estimate**	**SE**	**df**	**t.ratio**	**p.value**
	Control - High	0.11	0.04	111	2.55	0.05
	Control - Low	0.00	0.04	111	0.20	0.99
	Control - Medium	0.03	0.04	111	0.81	0.84
	High - Low	-0.10	0.04	111	-2.30	0.10
	High - Medium	-0.08	0.04	111	-1.75	0.29
	Low - Medium	0.02	0.04	111	0.59	0.93
**Ache**	**contrast - Copper concentration**	**estimate**	**SE**	**df**	**t.ratio**	**p.value**
	Control - High	4.12	1.70	6	2.42	0.11
	Control - Low	-1.02	1.70	6	-0.59	0.82
	High - Low	-5.14	1.70	6	-3.02	0.05
**N larvae eaten**	**contrast - Copper concentration**	**estimate**	**SE**	**df**	**t.ratio**	**p.value**
	Control - High	0.80	1.33	36	0.60	0.93
	Control - Low	0.30	1.33	36	0.22	0.99
	Control - Medium	1.10	1.33	36	0.83	0.83
	High - Low	-0.50	1.33	36	-0.37	0.98
	High - Medium	0.30	1.33	36	0.22	0.99
	Low - Medium	0.80	1.33	36	0.60	0.93
**Length of larvae**	**contrast - Copper concentration**	**estimate**	**SE**	**df**	**t.ratio**	**p.value**
	Control - High	3.30	0.04	111	67.37	<.0001
	Control - Low	0.82	0.04	111	16.90	<.0001
	Control - Medium	2.29	0.04	111	48.00	<.0001
	High - Low	-2.48	0.04	111	-49.78	<.0001
	High - Medium	-1.01	0.04	111	-20.64	<.0001
	Low - Medium	1.47	0.04	111	30.26	<.0001

p-value = significant. (Control= 0 μg L^{-1} , Low= 125 μg L^{-1} , Medium= 250 μg L^{-1} , High =500 μg L^{-1})

Table S3. pairwise comparisons as post-hoc tests (t-test) of the second generation *Culex pipiens* after seven days of copper exposure.

Generation 2						
**Distance**	**contrast - Copper concentration**	**estimate**	**SE**	**df**	**t.ratio**	**p.value**
	Control - Low	2.28	0.68	78	3.35	**0.00**
	Control - Medium	2.13	0.67	78	3.21	**0.01**
	Low - Medium	-0.15	0.67	78	-0.23	0.97
**Velocity**	**contrast - Copper concentration**	**estimate**	**SE**	**df**	**t.ratio**	**p.value**
	Control - Low	0.63	0.23	78	2.82	**0.02**
	Control - Medium	0.55	0.22	78	2.49	**0.04**
	Low - Medium	-0.09	0.22	78	-0.40	0.91
**Ache**	**contrast - Copper concentration**	**estimate**	**SE**	**df**	**t.ratio**	**p.value**
	Control - High	7.35	2.28	6	3.23	**0.04**
	Control - Low	-2.27	2.28	6	-0.99	0.61
	High - Low	-9.62	2.28	6	-4.22	**0.01**
**N larvae eaten**	**contrast - Copper concentration**	**estimate**	**SE**	**df**	**t.ratio**	**p.value**
	Control - High	-1.4	1.09	36	-1.28	0.58
	Control - Low	-0.1	1.09	36	-0.09	0.99
	Control - Medium	-1	1.09	36	-0.92	0.79
	High - Low	1.3	1.09	36	1.19	0.64
	High - Medium	0.4	1.09	36	0.37	0.98
	Low - Medium	-0.9	1.09	36	-0.82	0.84
**Length of larvae**	**contrast - Copper concentration**	**estimate**	**SE**	**df**	**t.ratio**	**p.value**
	Control - Low	12.50	0.39	78	31.72	<.0001
	Control - Medium	33.90	0.39	78	88.13	<.0001
	Low - Medium	21.40	0.39	78	55.56	<.0001

Table S4. Effect of copper concentrations, duration of predation by immature Libellulidae dragonflies, and their interaction, on the number of *Culex pipiens* larvae eaten after seven days of copper treatment. Two generations were exposed to several concentrations of copper sulphate or were unexposed. Effect of the random factor is not shown. p-values and Wald Chi-squared statistic (in parentheses) for each variable. Significance is indicated by *** $p < 0.001$.

		Generation 1	Generation 2
		N larvae eaten	N larvae eaten
Variables	Df	*p*	*p*
Duration of predation	1	**< 0.001 (34.60) *** **	**< 0.001 (32.00) *** **
Copper concentration	3	0.662 (1.59)	0.431 (2.75)
Duration × copper concentration	3	0.556 (2.08)	0.759 (1.18)
